# The Trojan Female Technique for pest control: a candidate mitochondrial mutation confers low male fertility across diverse nuclear backgrounds in *Drosophila melanogaster*

**DOI:** 10.1111/eva.12297

**Published:** 2015-08-26

**Authors:** Damian K Dowling, Daniel M Tompkins, Neil J Gemmell

**Affiliations:** 1 School of Biological Sciences, Monash UniversityClayton, Vic., Australia; 2 Landcare ResearchDunedin, New Zealand; 3 Allan Wilson Centre for Molecular Ecology and Evolution, Department of Anatomy, University of OtagoDunedin, New Zealand

**Keywords:** intergenomic conflict, male infertility, male-harming mutations, mito-nuclear, mother's curse, mtDNA, pest control, sterile male technique

## Abstract

Pest species represent a major ongoing threat to global biodiversity. Effective management approaches are required that regulate pest numbers, while minimizing collateral damage to nontarget species. The Trojan Female Technique (TFT) was recently proposed as a prospective approach to biological pest control. The TFT draws on the evolutionary hypothesis that maternally inherited mitochondrial genomes are prone to the accumulation of male, but not female, harming mutations. These mutations could be harnessed to provide trans-generational fertility-based control of pest species. A candidate TFT mutation was recently described in the fruit fly, *Drosophila melanogaster*, which confers male-only sterility in the specific isogenic nuclear background in which it is maintained. However, applicability of the TFT relies on mitochondrial mutations whose male-sterilizing effects are general across nuclear genomic contexts. We test this assumption, expressing the candidate TFT-mutation bearing haplotype alongside a range of nuclear backgrounds and comparing its fertility in males, relative to that of control haplotypes. We document consistently lower fertility for males harbouring the TFT mutation, in both competitive and noncompetitive mating contexts, across all nuclear backgrounds screened. This indicates that TFT mutations conferring reduced male fertility can segregate within populations and could be harnessed to facilitate this novel form of pest control.

## Introduction

Pests represent an ongoing threat, both to global biodiversity and the economic sustainability of agricultural industries (Pimentel et al. 2005). Invasive pest species can exert their effects via several ecological pathways, for example by introducing new pathogenic diseases into populations that were previously uninfected, via direct predatory behaviour, competition over resources and ecological niche spaces with native species counterparts, and by direct destruction of economically valuable resources intended for human consumption (Stenseth et al. 2003; Pimentel et al. 2005; Medina et al. 2011). As such, pest species are often the target of costly programs that seek to either eradicate their existence from particular areas or regulate their numbers to levels at which ecosystem or economic sustainability is upheld (Howarth 1991; Courchamp et al. 2003; Stenseth et al. 2003; Bergstrom et al. 2009).

Traditionally, pest management has focussed on conventional approaches that seek to directly remove target pest species from particular areas through the use of pesticides, herbicides, poison baits or manual culling. These approaches are usually expensive because they require ongoing and labour-intensive attention. Furthermore, application of poisons can both affect nontarget native species and result in some level of environmental contamination (Bergstrom et al. 2009; Warburton et al. 2012). Consequently, attention has turned to other forms of pest control that might offer longer term management solutions at lower prospective costs and that are less likely to result in collateral damage to other nontarget species or adverse environmental outcomes (Campbell et al. 2015).

Fertility control of pest species provides a theoretically and practically plausible approach to pest management (Courchamp and Cornell 2000; Cowan et al. 2002), but the development of such approaches has been slow (Tompkins 2007; Arthur et al. 2009; Cross et al. 2011). The most successful form of fertility control implemented to date has focussed on the manual sterilization of large numbers of the target pest species prior to their release into the focal population that is in need of control. This is the sterile male technique (SMT), commonly applied to invertebrate pests where males can be irradiated *en masse* at doses that render their gametes inviable but which have little effect on reproductive behaviour (Dyck et al. 2005; Alphey et al. 2010). Females that mate with irradiated males subsequently produce few or no offspring. While the SMT has proven successful at controlling invertebrate pests when implemented at large scales (Alphey et al. 2010), it requires large numbers of sterile males be produced and released over sequential generations and seasons. The time and labour costs of achieving this are prohibitive in many cases, such as when applied to vertebrate pests or pest management solutions in developing countries (Vreysen et al. 2007).

To address such issues, possible variants of the SMT, which entail some form of genetic modification that achieves heritable trans-generational male sterility in the pest species, are being pursued. These include Release of Insects carrying a Dominant Lethal (RIDL) (Thomas et al. 2000), daughterless carp (Thresher et al. 2002) and the Trojan Y chromosome (Gutierrez and Teem 2006). Most recently, the Trojan Female Technique (TFT) was proposed, based on the idea that male-sterilizing mutations in the mitochondrial DNA may be harnessed to provide a self-sustaining, multigenerational form of the SMT (Gemmell et al. 2013).

The TFT is based on the evolutionary hypothesis that mitochondrial genomes are prone to accumulate mutations that reduce male fitness without having parallel effects on female fitness (Frank and Hurst 1996). Maternal inheritance of mitochondria results in a sex-specific selective sieve on the mitochondrial DNA (mtDNA), because maternal inheritance means that mtDNA sequences are only directly screened by natural selection when carried by females. Therefore, if a mutation in the mtDNA arises that is benign (or nearly so) in its effects on female function, it can be maintained within a population even when this same mutation is associated with low male fitness (Frank and Hurst 1996; Gemmell et al. 2004; Beekman et al. 2014). These evolutionary predictions have been confirmed by population genetic models (Frank and Hurst 1996), and the existence of male-biased mtDNA mutations has also been empirically substantiated in fruit flies, *Drosophila melanogaster* (Innocenti et al. 2011; Camus et al. 2012), mice (Nakada et al. 2006), and by association in European hares, *Lepus europaeus* (Smith et al. 2010). Moreover, if mtDNA mutations were to arise that were overtly sexually antagonistic in effect – benefitting females, while harming males – these would be expected to be under strong positive selection, despite their negative effects on males, as a result of maternal transmission of the mtDNA (Unckless and Herren 2009; Innocenti et al. 2011). In the absence of demographic effects such as inbreeding or kin selection (Wade and Brandvain 2009), such sexually antagonistic mtDNA mutations would be predicted to move quickly towards fixation in a population (Unckless and Herren 2009; Innocenti et al. 2011); however, while such mutations are theoretically predicted, they have yet to be empirically substantiated. In theory, these male-harming mtDNA mutations, be they female-benign or female-beneficial, represent ‘candidate TFT mutations’ that could be harnessed to provide self-sustaining trans-generational fertility-based control of target pest species (Gemmell et al. 2013).

The TFT stands on robust theoretical ground. Population growth models have validated the potential capacity of the technique to achieve effective population control of target pest species over a wide range of target life histories (Gemmell et al. 2013). While the approach is predicted to be most effective when applied to pest species with high turnover rates and low rates of multiple mating, its additive nature means that multiple releases are predicted to control even species characterized by low turnover and high levels of multiple mating (Gemmell et al. 2013). However, while theoretical models indicate the promise of the TFT for pest control, the ability to harness candidate TFT mutations for pest population control hinges on two key criteria; first that mutations that reduce male, but not female, fertility exist in the mtDNA sequence, and second that the effects of such mutations are additive across a large range of nuclear genomic and environmental contexts (i.e. not contingent on mito-nuclear epistasis or genotype-by-environment effects).

Addressing the first criterion, experimental substantiation of the existence of mtDNA mutations that specifically affect male – but not female – fertility outcomes now exists in fruit flies (Clancy et al. 2011; Innocenti et al. 2011; Yee et al. 2013), mice (Nakada et al. 2006) and hares *Lepus europaeus* (Smith et al. 2010). Some of the best metazoan examples come from *D. melanogaster*, in which there is experimental evidence that mtDNA haplotypes harbour male-biased mutation loads that confer widespread interference of gene expression across the male, but not female, nuclear transcriptome. The nuclear genes most sensitive to these male-biased mitochondrial mutation loads are those entwined in male-specific reproductive functions (Innocenti et al. 2011). Indeed, a candidate TFT mutation was recently identified in *D. melanogaster*, which confers male-only sterility in one isogenic fly strain (Clancy et al. 2011) and reduced fertility in another (Yee et al. 2013). Females that carry this mutation, however, remain fully fertile (M. F. Camus and D. K. Dowling, personal communication). The mutation was identified in a naturally occurring mtDNA haplotype derived from Brownsville, Texas USA (Clancy 2008). It is a nonsynonymous mutation in the mt-CYTB gene of respiratory complex III, which results in an amino acid transition (Ala278 → Thr). This threonine variant at amino acid site 278 in mt-CYTB, while rare, has been identified in a range of other taxa, both invertebrates (heartworms, crickets) and vertebrates, including frogs, gulls and howler monkeys (Clancy et al. 2011). Thus, this candidate mutation could be of broad applicability in the development of the TFT.

The second criterion has yet to be empirically addressed. When it comes to the candidate mt-CYTB mutation described above, the associated male-infertility phenotype associated with this mutation remains uncharacterized outside of *D. melanogaster*. Even for *D. melanogaster* it is unknown whether the male-infertility phenotype associated with this mutation is specific to the standard nuclear backgrounds in which previous experiments have been conducted (Clancy et al. 2011; Yee et al. 2013) and thus dependent on a mito-nuclear interaction. Suitability of this mutation for the TFT, however, is contingent on the male-harming effects being additive across a range of nuclear backgrounds, such that the males harbouring this mutation are in- or subfertile regardless of their associated nuclear genotype (Gemmell et al. 2013).

Here we test this criterion by expressing the Brownsville mtDNA haplotype, which harbours the candidate TFT mutation, alongside numerous nuclear genomic backgrounds sourced from five different populations, comparing the fertility of males bearing the TFT mutation to males possessing each of two putatively healthy control haplotypes. Male fertility was assessed in two environmental mating contexts, both noncompetitively whereby TFT-bearing males were provided with sole access to virgin females, and competitively whereby the sperm of TFT-bearing males was in competition with sperm from a rival male for fertilizations.

## Methods

### Creation of mitochondrial strains

We compared three naturally occurring mitochondrial haplotypes, originally derived from different worldwide *D. melanogaster* populations. These populations were Brownsville Texas, USA (Rand et al. 1994); Puerto Montt, Chile (Calboli et al. 2003) and Zimbabwe (Ballard and Kreitman 1994). Each of the haplotypes was placed alongside an isogenic nuclear background (*w*^*1118*^, Bloomington stock no. 5905) by Clancy (2008) to create ‘mitochondrial’ strains. We obtained these strains in 2007, and created duplicates of each, which have since been propagated independently of each other. Since 2007, we have put the *w*^*1118*^ strain through a further 75 generations of full-sibling mating to guarantee its isogenicity and have likewise backcrossed virgin females of each mitochondrial strain duplicate to males of the *w*^*1118*^ background over 75 generations. Using duplicates of each of the three mitochondrial strains enables us to disentangle true mitochondrial genetic effects from those linked to any residual nuclear allelic variance or environmental sources of variance.

Our candidate TFT mutation, the Brownsville haplotype (BRO), is completely male sterile but female fertile when expressed alongside the *w*^*1118*^ nuclear background (Clancy et al. 2011). Male sterility can be traced to a single SNP in the mt-CYTB gene. The other two haplotypes, Puerto Montt (PUE) and Zimbabwe (ZIM), are putatively healthy in both males and females, and act as control haplotypes in our experiments.

We also generated standardized heterozygote brown-eyed flies to use as tester females and tester males in our competitive fertility assays. These were created by nonreciprocally crossing two near-isogenic lines [derived from a replica of the LH_M_ population fixed for an autosomal recessive mutation that encodes brown eyes (Friberg and Dowling 2008)]. For our experiments, we used the F_1_ offspring, exhibiting high levels of genomewide heterozygosity per individual, but near identical genotypes across individuals of a given sex.

Flies were reared in 40 mL plastic vials on a potato-yeast-dextrose-agar substrate, with *ad libitum* live yeast added to the surface. Unless otherwise stated, egg density per vial was trimmed to 150 eggs to control for larval density effects. All strains were kept at a constant 25°C with a 12:12 h light:dark cycle. Adult flies were transferred to fresh vials at least once every 2 days. Flies were lightly anaesthetized with CO_2_ when sorting virgin males from females.

### Experimental design

We conducted crosses between females of the mitochondrial strains and males of five other laboratory-reared strains and used the F_1_ sons as the focal males in the experiments (Table1). Each focal male possessed one of the three mtDNA haplotypes (inherited from the mother), which was expressed alongside a haploid nuclear copy of the *w*^*1118*^ strain (inherited from the mother) and a haploid nuclear copy of one of five laboratory strains (inherited from the father). Three of these strains are outbred panmictic populations that are maintained at large effective population sizes: Coffs Harbor (CH) from NSW, Australia (Williams et al. 2012; Dowling et al. 2014); Dahomey (DAH) collected in 1970 from Benin, Africa (Partridge and Andrews 1985); LH_M_ (LHM) collected in 1991 by Larry Harshman from California, USA (Rice et al. 2005; Friberg and Dowling 2008). The other two strains are the isofemale lines Brownsville and Puerto Montt (Clancy 2008), from which the BRO and PUE mtDNA haplotypes were sourced, which have been subjected to 17 generations of full-sibling pair mating to ensure near-isogenicity.

**Table 1:** Crossing scheme involving females of mitochondrial strains and males from five other laboratory-reared strains, and the resulting genotypes of the F_1_ focal males. The mitochondrial strains are depicted in the left-hand column and comprise the Brownsville haplotype (BRO), which harbours the candidate TFT mutation, and two control haplotypes originally sourced from Puerto Montt (PUE) and Zimbabwe (ZIM). The laboratory strains that contributed haploid nuclear genomes to the focal males were comprised of three outbred strains and two isogenic lines. The outbred strains were sourced from Coffs harbour (CH), Dahomey (DAH) and LH_M_ (LHM), the isogenic strains from Brownsville (BRO) and Puerto Montt (PUE). Each cross was independently replicated, and this was possible because each mitochondrial strain had been maintained in duplicate for 7 years prior to these experiments. The 15 focal genotypes of resulting focal males (that were assayed for fertility) are denoted in the table, with the mtDNA haplotype inherited from the mother, one haploid copy of the *w*^*1118*^ nuclear genome inherited from the mother (from the mitochondrial strains) and the other haploid copy of the nuclear genome from the laboratory-strain fathers

Maternal contribution	Paternal contribution				
	Outbred laboratory strains			Isogenic nuclear strains	
Mitochondrial strains	Coffs harbour (CH)	Dahomey (DAH)	LH_M_ (LHM)	BROWNSVILLE (BRO)	PUERTO MONTT (PUE)
TFT – BRO	BRO mtDNA *w*^*1118*^: CH	BRO mtDNA *w*^*1118*^: DAH	BRO mtDNA *w*^*1118*^: LHM	BRO mtDNA *w*^*1118*^: BRO	BRO mtDNA *w*^*1118*^: PUE
Control 1 – PUE	PUE mtDNA *w*^*1118*^: CH	PUE mtDNA *w*^*1118*^: DAH	PUE mtDNA *w*^*1118*^: LHM	PUE mtDNA *w*^*1118*^: BRO	PUE mtDNA *w*^*1118*^: PUE
Control 2 – ZIM	ZIM mtDNA *w*^*1118*^: CH	ZIM mtDNA *w*^*1118*^: DAH	ZIM mtDNA *w*^*1118*^: LHM	ZIM mtDNA *w*^*1118*^: BRO	ZIM mtDNA *w*^*1118*^: PUE

While the focal sons resulting from crosses between the mitochondrial strains and outbred laboratory strains inherited an identical haploid copy of the *w*^*1118*^ nuclear genome from their mothers, the copy they received from their fathers comprised a population representative (i.e. CH, DAH or LH_M_) but nonetheless unique nuclear haploid genotype. In contrast, offspring produced from crosses involving the isogenic nuclear strains resulted in completely standardized nuclear genotypes, such that all sons from a given cross shared a near identical genotype (Table1). This allowed us to probe the significance of mitochondrial–nuclear interactions at two levels; first in a very controlled setting in which each mitochondrial haplotype was screened against two different nuclear genetic backgrounds in the absence of any segregating nuclear allelic variance; second in a more realistic setting in which mtDNA haplotypes were screened against three population-representative nuclear genomic contexts, each in the presence of an abundance of segregating nuclear allelic variance.

To achieve the crosses, virgin females were collected from each mitochondrial strain and stored in groups of 10. When 3 days old, each group was transferred to a fresh vial containing 10 equivalently aged males from one of the five strains, with all mitochondrial strain × laboratory strain cross-combinations carried out. This diallel of crosses was replicated in full with females of each of the mitochondrial strain duplicates. After 24 h, each group of females and males was transferred to a new vial, and the females provided with a 24 h opportunity to lay eggs. Egg density per vial was kept to between 40 and 80 eggs. Ten days later, eclosing virgin focal males were collected from these vials. These males were assigned to one of the two fertility assays at 3 days of age, measuring fertility either when mating with a female in the absence of mating competition, or in the presence of competition with sperm from a rival male.

### Noncompetitive fertility assay

Each focal male was provided with three consecutive 24-h mating opportunities. During each 24-h period, the male was placed in a vial containing two 3-day-old virgin ‘tester’ females. Thus, each male was given the opportunity to mate with six tester females over 72 h. Following each 24-h period, each pair of tester females was transferred to a new vial for a subsequent 24-h period. Thus, each tester female was given 48 h to lay eggs over two vials. Male fertility was scored 14 day later, as the number of offspring eclosing from their pupal cases. Assays were run in separate blocks for the inbred versus the outbred nuclear strains.

### Competitive fertility assay

Each focal male was provided an opportunity to induce a once-mated female to re-mate, and his resulting paternity was then assigned based on the eye colour of the offspring. To start the assay, each virgin tester female was provided with a 150-min mating opportunity with a virgin tester male. Our previous observations with these heterozygous standard genotype flies indicated that virtually all females would mate once and only once within this timeframe (Yee et al. 2013). The tester male was then discarded and a focal male added to the mating vial. Focal males were then given 29 h to induce the tester females to re-mate. Tester females were then transferred to a new vial and allowed to lay eggs for 20 h. The eclosing offspring of this vial were scored for eye colour 13 day later. Tester flies were fixed for an autosomal recessive mutation that encodes brown eyes, and thus the presence of wild-type (red eyed) offspring indicated individuals sired by the focal males, and brown-eyed offspring indicated those sired by the tester males. Male competitive fertility was thus denoted by the proportion of red-eyed offspring within the vial. Each of the original mating vials was retained to ensure that the first matings, between the tester females and males, had been successful and resulted in viable brown-eyed offspring. In addition to being run separately from one another, the assays involving the outbred versus the inbred nuclear strains were each split into two blocks.

### Statistical analysis

We fitted multilevel linear and generalized linear models to the fertility data, using the *lme4* package (Bates et al. 2014) in R 3.0.3 (R Core Team 2014). Response variables were noncompetitive male fertility (total offspring production per male) and competitive male fertility (a binomial vector comprising the number of offspring sired by the focal male and the number sired by the tester male). Separate models were run for the experiments involving the outbred versus the inbred nuclear strains. Noncompetitive fertility was modelled using a Gaussian distribution and competitive fertility a binomial distribution and logit link. Fixed effects in all analyses were the mtDNA haplotype (BRO, PUE, ZIM), the haploid nuclear background (BRO or PUE for the isogenic strain analyses; COFF, DAH or LHM for the outbred strain analyses), with interactions between mtDNA haplotype and haploid nuclear background also included. Random effects were the identity of the vial in which the focal males had been reared, which was nested within the identity of the cross-replicate (each particular combination of mitochondrial strain × laboratory strain cross had been replicated independently). Analyses of competitive fertility also included block number as a random effect and an observation-level random effect because the binomial models were over-dispersed (Browne et al. 2005). Significance of effects was assessed using Type III Wald *F* tests with Kenward-Roger correction of degrees of freedom (Stroup 2015) in the *car* package (Fox and Weisberg 2011) for the noncompetitive fertility models and Type III Wald chi-square tests for the competitive fertility models.

## Results

**Figure 1:**
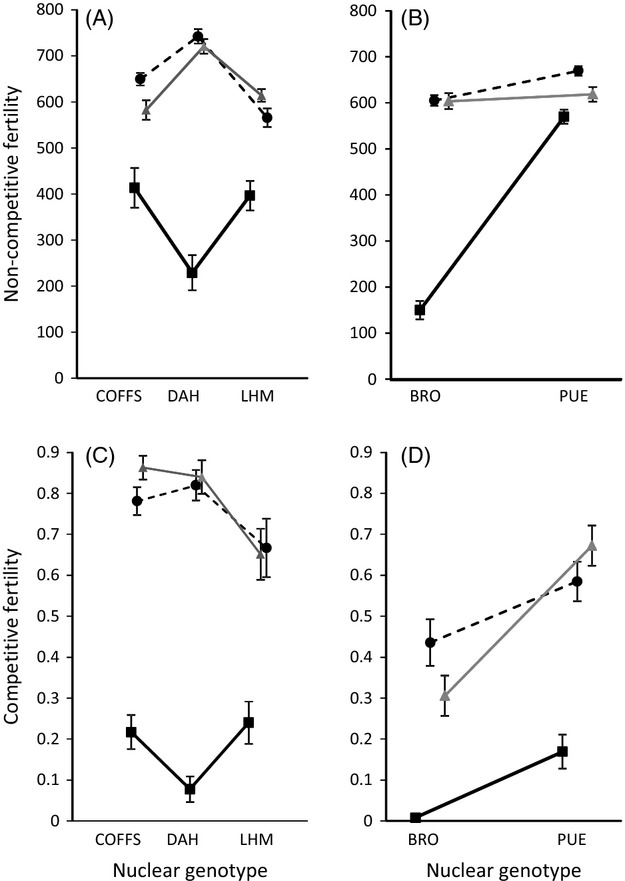
Fertility of the three mtDNA haplotypes when expressed alongside haploid nuclear genomes from outbred (A, C) and isogenic (B, D) strains. Noncompetitive fertility (A, B) represents the number of offspring sired (mean offspring number per genotype ± 1 SE) by focal males of each mito-nuclear genotype. Competitive fertility (C, D) represents a measure of fertility (mean proportion of offspring sired per genotype ± 1 SE) when focal males were placed with standard females that had previously mated once to a rival male of standardized genotype. The haploid nuclear genotype is denoted on the horizontal axis, and the three mtDNA haplotypes are delineated by the symbol shape and line format. The BRO haplotype, which harbours the TFT mutation, is represented by closed black squares, connected by solid black lines; the PUE haplotype is represented by closed black circles connected by dashed black lines, and the ZIM haplotype is represented by closed grey triangles connected by solid grey lines.

The BRO haplotype, which harbours the candidate TFT mutation, consistently exhibited lower noncompetitive fertility relative to controls when expressed alongside haploid copies of nuclear genotypes sourced from the three outbred nuclear strains (*F*_2,8.90_ = 17.1, *P* < 0.001). However, the magnitude of this effect was contingent on an interaction with the nuclear background (*F*_4,8.99_ = 11.9, *P* = 0.001), with BRO being particularly poorly performing when expressed alongside DAH (Fig.1A, Table S1). Similarly, the BRO haplotype consistently exhibited lower noncompetitive fertility relative to controls, when expressed alongside haploid copies of nuclear genomes provided by the isogenic nuclear strains (*F*_2,5.66_ = 297.5, *P* < 0.001), and again this mitochondrial haplotypic effect was influenced by the nuclear background (*F*_2,6.01_ = 101.5, *P* < 0.001). Fertility of the BRO haplotype was markedly lower when expressed alongside the BRO nuclear genome than alongside PUE (Fig.1B, Table S2).

The BRO haplotype also consistently conferred lower competitive fertility than the other mitochondrial haplotypes, when expressed alongside haploid genotypes sourced from the three outbred nuclear strains (*χ*^2^ = 76, df = 2, *P* < 0.001). The magnitude of this effect was influenced by the nuclear background (*χ*^2^ = 35.6, df = 4, *P* < 0.001), with the BRO haplotype again performing worst when expressed alongside the DAH nuclear genome (Fig.1C, Table S3). Likewise, the BRO haplotype consistently exhibited lower competitive fertility when expressed alongside nuclear backgrounds provided by the isogenic nuclear strains (*χ*^2^ = 77.6, df = 2, *P* < 0.001), and this mitochondrial effect was influenced by the nuclear background (*χ*^2^ = 13.49, df = 2, *P* = 0.001). Fertility of the BRO haplotype was again markedly lower when expressed alongside the BRO nuclear genome than alongside PUE (Fig.1D, Table S4).

## Discussion

We found consistent mitochondrial genetic effects on noncompetitive and competitive components of male fertility. However, the magnitude of these effects was not entirely additive, with mito-nuclear interactions contributing to the expression of male fertility. In particular, the degree of fertility reduction associated with the BRO haplotype, which harbours the candidate TFT mutation, clearly depended on the nuclear background alongside which it was expressed. These results indicate that polymorphisms within the mitochondrial genome, including the TFT mutation, interact epistatically with those within the nuclear genome to shape male fertility. Importantly, however, mean fertility of the BRO haplotype was substantially reduced relative to controls (decreases of between 29% and 69% for all but one nuclear background in the noncompetitive fertility assay and decreases of between 71% and 97% across all mito-nuclear combinations in the competitive assay, Fig.1). In one instance, the BRO haplotype exhibited only a modest decrease in noncompetitive fertility relative to the controls (a decrease of 8% when expressed alongside the PUE nuclear genome). We note, however, that this epistatic mito-nuclear combination nonetheless translated into a substantial reduction in competitive fertility (71% decrease). These findings are key to the feasibility of the TFT.

There are several possible mechanisms that could underpin the low competitive fertility of the TF males. However, we can eliminate the possibility that the low TF male competitive fertility was driven by low egg-to-adult viability of the eggs that they fertilized. If low viability were the principal driver, then we would have expected to observe lower total offspring production, in the competitive fertility assays, for tester females who were assigned to the TF matings than those assigned to the control. There were clearly no differences in the number of offspring produced per female, resulting from the 20-h period in which these females were able to oviposit, for mating trials involving control males (mean_outbred strains_ = 41.4 ± 1, mean_isogenic strains_ = 38.6 ± 1.6), relative to those involving TF males (mean_outbred strains_ = 40.4 ± 1.4, mean_isogenic strains_ = 41.4 ± 1.6). This indicates that processes occurring prefertilization decided the reduced competitive fertility outcomes of TF males. Furthermore, while instances of zero paternity assigned to the TF males may well have occurred due to their failure to induce tester females into mating, there were many cases in which the TF males unambiguously mated with these females (i.e. many non-zero paternity values). In such cases, fertilizations, under sperm competition, were heavily biased against the TF males (mean paternity of TF males = 27.3 ± 0.02%), indicating that the sperm produced by TF males was clearly outcompeted in competition with sperm of the tester males.

The TFT is based on the application of male-harming mitochondrial mutations, which depress male fertility but have little or no concomitant effects on the fertility of females. Population growth models have indicated that a TFT mutation could achieve more effective pest regulation than the SMT over a wide range of target life histories (Gemmell et al. 2013), including in cases where population turnover rates are low and multiple mating rates high (Gemmell et al. 2013). However, while population models indicate the promise of the TFT for pest control, its practicability hinges on whether candidate TFT mutations (conferring general reductions in male fertility while having little or no impact on females) exist in eukaryote mitochondrial genomes and can be harnessed to achieve pest control. Promisingly, the general level of fertility reduction documented here is well within the predicted range of necessary effect sizes for the TFT approach to pest control to be a valid proposition (Tompkins 2015).

Whether the candidate TFT mutation that we studied here, which is nested within the BRO haplotype, can be harnessed to achieve efficient population regulation depends on several criteria, each of which might stand in the way of the development of this technique, and each of which is therefore in need of further testing. The first criterion, and focus of our current study, is that the male-fertility effects associated with this candidate mutation are general across a range of genotypic and environmental contexts. Addressing this, we confirmed that the BRO haplotype was consistently poorly performing relative to controls across numerous nuclear genetic backgrounds sourced from five different global localities and across two environmental mating contexts. This suggests that the additive effects of the TFT mutation will generally outweigh those due to mito-nuclear epistasis or gene-by-environment interactions. However, it will be valuable to conduct studies that further scrutinize the generality of our findings, for instance by testing the TFT mutations across a range of abiotic environmental conditions such as thermal or nutritional gradients.

All of the fertility assessments associated with the BRO haplotype to date, including in the current study, have been conducted in the presence of at least one haploid copy of the *w*^*1118*^ strain (Clancy et al. 2011; Innocenti et al. 2011; Yee et al. 2013). When expressed alongside a diploid *w*^*1118*^ nuclear background, the BRO haplotype is completely sterile (Clancy et al. 2011), while when expressed alongside other nuclear backgrounds that consist of one distinct nuclear haploid genome and one copy of *w*^*1118*^, the BRO haplotype exhibits reduced fertility. It is possible that this reduced fertility is therefore traceable to a specific interaction between the TFT mutation and a polymorphism specific to the *w*^*1118*^ background (i.e. a mito-nuclear interaction involving *w*^*1118*^). This possibility can be tested by expressing the BRO haplotype alongside nuclear genomic backgrounds that lack any contribution from *w*^*1118*^ to determine whether the reduced fertility is upheld in the absence of *w*^*1118*^, and this should be addressed in future research efforts. That said, we note that this candidate TFT is rare in metazoans; while it has been identified in a variety of taxa, this equates to only 19 mtDNA sequences in total (Clancy et al. 2011). It is plausible its rarity is linked to low performance generally.

Another criterion is the feasibility of using the candidate mutation studied here (which is located in the mt-CYTB gene and which alters the amino acid sequence) in other species, including those that are targets of pest control efforts. In particular, it would be desirable that this mutation should confer a similar male-sterilizing effect in those systems. The candidate TFT mutation tested here has already been detected in a range of taxa, from heartworms to seagulls (Clancy et al. 2011). Experimental studies can also scrutinize segregating mitochondrial haplotypes for the presence of other male-harming mtDNA mutations, via mitochondrial replacement techniques that are able to partition mitochondrial from nuclear genetic effects on phenotypic expression (Dowling et al. 2008; Reinhardt et al. 2013). These can be combined with mitochondrial genomewide association studies to search for other candidate mutations. A recent study of mitochondrial genetic variance for longevity in *D. melanogaster* presented evidence that mtDNA haplotypes may well harbour numerous male-harming mtDNA mutations of minor effect, rather than a few of major effect (Camus et al. 2012). If this is the case, ‘whole haplotypes’ that are poor performers in males, due to numerous male-biased mtDNA mutations of minor effect, could potentially become the targets of TFT efforts.

A third criterion is that the male-harming mutations must be strongly male-biased, with limited or no positive pleiotropy in fertility across the sexes. Evolutionary theory suggests that such mutations may actually often be overtly sexually antagonistic in their effects, exhibiting negative pleiotropy and increasing female fitness at the expense of males (Bonduriansky and Chenoweth 2009). It is well known that sexually antagonistic mutations that confer cytoplasmic male sterility are often harboured within the mitochondrial genomes of hermaphroditic plants. These mutations effectively convert the hermaphrodite to females, and consequently, these mitochondrial genomes rapidly accumulate under positive selection (Budar and Fujii 2012). All evidence collated to date indicates that the TFT mutation under study here is heavily male-biased in its harm (Clancy 2008; Clancy et al. 2011; Innocenti et al. 2011). However, it is plausible that the mutation may exert some pleiotropic effect on females. If this effect was beneficial to some component of female fitness, it would mean that the TFT mutation would conceivably accumulate under positive selection (Unckless and Herren 2009), which would further enhance its potential to achieve population regulation.

Evidence is also accumulating that explicitly male-harming mtDNA mutations can be offset by nuclear modifiers in *Drosophila* (Sackton et al. 2003; Yee et al. 2013), and the existence of nuclear adaptations that reverse mtDNA-invoked male-sterilization in hermaphroditic plants is well established (Budar and Fujii 2012). The introduction of a male-sterilizing TFT into a pest population will in theory place strong selection for compensatory nuclear modifiers. But, applicability of male-harming mtDNA mutations to the TFT assumes that pest populations do not quickly evolve such nuclear counter-responses. To this end, the compensatory mutations should already be present in the standing nuclear allelic variance, if the nuclear genomes are to quickly respond. We found that the effects of the candidate TFT mutation were indeed modulated by the nuclear background, suggesting that such nuclear modifiers might well exist. However, on the other hand, if the TFT mutations achieve swift population regulation in line with the expectations of mathematical models (Gemmell et al. 2013), resulting in sharp reductions in effective population sizes (*N*_e_), the scope for nuclear compensation might be low. This is because the efficacy of selection acting on potential modifier alleles will be reduced at low *N*_e_ and, furthermore, the initial rapid bottleneck of standing genetic variance resulting from TF-induced population regulation (particularly if combined with manual culling-based or traditional SMT strategies of regulation) could well purge rare modifier alleles that were segregating in the pest population. Under this scenario, frequencies of nuclear alleles segregating within the pest populations could be dictated primarily by genetic drift, favouring the effectiveness of the TFT approach. The degree to which nuclear counter-adaptation is likely to impede the efficacy of the TFT remains an open question and requires experimental attention through population regulation studies. Finally, we note that the TFT effects identified in this study were detected under controlled and benign laboratory conditions. It thus remains to be tested whether such effects will be upheld when applied to wild populations, where environmental (e.g. climatic) and demographic (age structures, densities and operational sex ratios) factors will exhibit much greater spatial and temporal heterogeneity.

Our experiments provide support for the general validity of the TFT and indicate that experiments could move on to determining whether the candidate TFT mutation studied here can effect efficient numerical regulation when introduced into focal populations. The mitochondrial genome currently remains locked from genome editing techniques that enable specific mutations to be inserted into it, or deleted from it, which constrains progress on the development of the TFT. If developed, it would provide the simplest means with which to insert candidate TFT mutations into focal pest species. However, conventional mutagenic approaches (e.g. EMS; St Johnston 2002) also provide promising alternative avenues for generating further candidate TFT mutations for assessment. Fortunately, the accumulating evidence suggests that male-harming mtDNA mutations are a natural component of the mutational landscape of animal and plant mitochondrial genomes (Beekman et al. 2014), and initial efforts will therefore focus on whether these can be harnessed to develop this novel form of biocontrol.

## Data archiving

Data available from the Dryad Digital Repository: http://dx.doi.org/10.5061/dryad.j1c4t.

## Supporting Information

**Table S1** Mixed model (REML) estimating effects of mtDNA haplotype and haploid nuclear genotype (sourced from outbred nuclear strains) on male non-competitive fertility. **Table S2.** Mixed model (REML) estimating effects of mtDNA haplotype and haploid nuclear genotype (sourced from isogenic nuclear strains) on male non-competitive fertility. **Table S3.** Generalized linear mixed model estimating effects of mtDNA haplotype and haploid nuclear genotype (sourced from outbred nuclear strains) on male competitive fertility. **Table S4.** Generalized linear mixed model (binomial error variance, logit link function) estimating effects of mtDNA haplotype and haploid nuclear genotype (sourced from inbred nuclear strains) on male competitive fertility.
