# Supplementary material for: The Trojan Female Technique for pest control: a candidate mitochondrial mutation confers low male fertility across diverse nuclear backgrounds in Drosophila melanogaster
Source: Evol Appl. 2015 Aug 26;8(9):871–80. doi: 10.1111/eva.12297 (PMC4610384; doi:10.1111/eva.12297)
Supplement: Supplementary file 1 — Table S1 Mixed model (REML) estimating effects of mtDNA haplotype and haploid nuclear genotype (sourced from outbred nuclear strains) on male non-competitive fertility. Table S2. Mixed model (REML) estimating effects of mtDNA haplotype and haploid nuclear genotype (sourced from isogenic nuclear strains) on male non-competitive fertility. Table S3. Generalized linear mixed model estimating effects of mtDNA haplotype and haploid nuclear genotype (sourced from outbred nuclear strains) on male competitive fertility. Table S4. Generalized linear mixed model (binomial error variance, logit link function) estimating effects of mtDNA haplotype and haploid nuclear genotype (sourced from inbred nuclear strains) on male competitive fertility. [file eva0008-0871-sd1.docx]

**The Trojan Female Technique for pest control: a candidate mitochondrial mutation confers low male fertility across diverse nuclear backgrounds and mating contexts in *Drosophila melanogaster***

**Electronic Supporting Information**

**Table S1.** Mixed model (REML) estimating effects of mtDNA haplotype and haploid nuclear genotype (sourced from outbred nuclear strains) on male non-competitive fertility. Fixed effects tested using a Type III Wald F test with Kenward-Roger degrees of freedom approximation.

| **Source of variance** | **F** | **DF** | **p** |
| --- | --- | --- | --- |
| ***Fixed effects*** |  |  |  |
| Intercept | 142.78 | 1, 6.02 | < 0.001 |
| mtDNA haplotype | 17.10 | 2, 8.90 | < 0.001 |
| Nuclear genotype | 10.76 | 2, 8.90 | 0.004 |
| mtDNA × Nuclear | 11.94 | 4, 8.99 | 0.001 |
| ***Random effects*** | ***SD*** |  |  |
| Vial ID | 46.9 |  |  |
| Cross replicate | 4.68 × 10^-9^ |  |  |
| Block | 23.2 |  |  |
| Residual | 114.1 |  |  |

**Table S2.** Mixed model (REML) estimating effects of mtDNA haplotype and haploid nuclear genotype (sourced from isogenic nuclear strains) on male non-competitive fertility. Fixed effects tested using a Type III Wald F test with Kenward-Roger degrees of freedom approximation.

| **Source of variance** | **F** | **DF** | **p** |
| --- | --- | --- | --- |
| ***Fixed effects*** |  |  |  |
| Intercept | 98.08 | 1, 5.51 | < 0.001 |
| mtDNA haplotype | 297.51 | 2, 5.66 | < 0.001 |
| Nuclear genotype | 366.78 | 1, 6.02 | < 0.001 |
| mtDNA × Nuclear | 101.48 | 2, 6.01 | < 0.001 |
| ***Random effects*** | ***SD*** |  |  |
| Vial ID | 0 |  |  |
| Cross replicate | 0 |  |  |
| Residual | 74.18 |  |  |

**Table S3.** Generalized linear mixed model estimating effects of mtDNA haplotype and haploid nuclear genotype (sourced from outbred nuclear strains) on male competitive fertility.

| **Source of variance** | **χ^2^** | **DF** | **p** |
| --- | --- | --- | --- |
| ***Fixed effects*** |  |  |  |
| Intercept | 3.88 | 1 | 0.048 |
| mtDNA haplotype | 75.95 | 2 | < 0.001 |
| Nuclear genotype | 22.70 | 2 | < 0.001 |
| mtDNA × Nuclear | 35.62 | 4 | 0.001 |
| ***Random effects*** | ***SD*** |  |  |
| Observation-level effect | 1.75 |  |  |
| Vial ID | 2.21 × 10^-1^ |  |  |
| Cross replicate | 1.90 × 10^-16^ |  |  |
| Block | 8.51 × 10^-1^ |  |  |

**Table S4.** Generalized linear mixed model (binomial error variance, logit link function) estimating effects of mtDNA haplotype and haploid nuclear genotype (sourced from inbred nuclear strains) on male competitive fertility.

| **Source of variance** | **χ^2^** | **DF** | **p** |
| --- | --- | --- | --- |
| ***Fixed effects*** |  |  |  |
| Intercept | 112.77 | 1 | < 0.001 |
| mtDNA haplotype | 77.61 | 2 | < 0.001 |
| Nuclear genotype | 30.50 | 1 | < 0.001 |
| mtDNA × Nuclear | 13.49 | 2 | 0.001 |
| ***Random effects*** | ***SD*** |  |  |
| Observation-level effect | 1.32 |  |  |
| Vial ID | 1.13 × 10^-5^ |  |  |
| Cross replicate | 0 |  |  |
